# Serum fibrinogen levels are positively correlated with advanced tumor stage and poor survival in patients with gastric cancer undergoing gastrectomy: a large cohort retrospective study

**DOI:** 10.1186/s12885-016-2510-z

**Published:** 2016-07-14

**Authors:** Xuefeng Yu, Fulan Hu, Qiang Yao, Chunfeng Li, Hongfeng Zhang, Yingwei Xue

**Affiliations:** Harbin Medical University Cancer Hospital, Haping Rd. #150, Harbin, 150040 Helongjiang Province People’s Republic of China; Harbin Medical University Public Health College, Harbin, 150081 Helongjiang Province People’s Republic of China

**Keywords:** Fibrinogen, Prognosis, Survival, Gastric cancer, Risk factor

## Abstract

**Background:**

Platelet and blood coagulation abnormalities frequently occur in cancer patients. Fibrinogen is an important hemostatic factor that regulates the hemostatic pathway. Hyperfibrinogenemia is increasing recognized as an important risk factor influencing cancer development and outcome. However, few reports have investigated the prognostic potential of fibrinogen for predicting the survival of gastric cancer (GC) patients. The primary aim of this study was to evaluate the usefulness of preoperative serum fibrinogen as a biomarker for predicating tumor progression and survival of patients with GC.

**Patients and methods:**

This retrospective study was conducted in GC patients who underwent gastrectomy from 2005 to 2007. Patient demographics, clinicopathological characteristics, preoperative plasma fibrinogen levels and median survival time (MST) were analyzed. Univariate and multivariate proportional hazard analysis of risk factors were used.

**Results:**

This study included 1196 patients (885 males and 311 females) with GC, more than half of whom had advanced GCs. Radical lymph node dissection was performed in 71.6 % of these patients. MST was 41.9 ± 32.4 months. Patient survival was significantly affected by family GC history (*p* <0.05), lymph node dissection mode (*p* <0.001), tumor size (≥5 cm; *p* <0.001), tumor location (*p* < 0.001), poor tumor differentiation (*p* <0.001), tumor histologic classification (*p* <0.001), extent of tumor invasion (*p* <0.001), number of metastatic lymph nodes (*p* <0.001), advanced stage of disease (*p* <0.001), extended operation duration (>150 min; *p* <0.001), higher operative bleeding volume (>200 ml; *p* <0.001), postoperative transfusion, preoperative serum fibrinogen levels, CEA levels and CA 19-9 levels (*p* <0.001). Multivariate analysis indicated that the independent prognostic factors significantly associated with poor survival included non-radical lymph node dissection, palliative lymph node dissection, multi-organ involvement, advanced TNM stages, poor tumor differentiation, higher preoperative serum fibrinogen levelsand higher CA19-9 levels.

**Conclusions:**

Serum fibrinogen levels are positively correlated with advanced tumor stages and poor survival in GC patients undergoing gastrectomy. Preoperative plasma fibrinogen levels are an independent risk factor for survival in these patients. Serum fibrinogen is a useful biomarker for patients with clinically advanced GC.

## Background

Gastric cancer (GC) is one of the most common malignancies and the third leading cause of cancer related death worldwide [1]. Early diagnosis of GC is very difficult, and the majority of GC cases are diagnosed during advanced stages with distant metastasis [2]. GC incidence and mortality are particularly high in Asia [2]. According to the 2012 estimations of the World Health Organization’s GLOBOCAN project, the age standardized rates (ASR) of GC incidence and mortality in Asia were 15.8 and 11.7 per 100,000, respectively [2].

Although advancements have been made in GC chemotherapy and local control, patient prognosis remains poor. The overall 5-year survival rate of patients with GC is estimated to be between 10 and 30 % in US and Europe. However, large regional differences exist between eastern and western nations. For instance, survival rates in Japan have been reported to range from 50 to 70 % [3]. The most prominent prognostic factors affecting the outcome and survival of GC patients are tumor related factors, including tumor size, lymph node metastasis, the degree of tumor cell differentiation, the extent of tumor invasion and the presence of distant metastasis [4–9]. Studies suggest that increasing tumor size, advanced TNM stages and poor differentiation are all important indicators of aggressive GC and predict worse outcome [10, 11].

It is increasingly being recognized that, in addition to tumor related factors, GC patient prognosis is also affected by operation and patient related factors [12]. For example, intraoperative blood loss and transfusion delay are associated with worse post-surgical patient outcomes [12]. Additionally, age, sex, inflammatory response, abnormal blood coagulation and comorbidity have also been correlated with poor survival and prognosis [13, 14].

Platelet and blood coagulation abnormalities occur frequently in cancer patients. Thrombocytosis is considered an important risk factor, and it is associated with poor GC prognosis [14]. Fibrinogen is a 340-kDa glycoprotein that is primarily produced by hepatic cells, and is an important clotting factor that helps regulate the hemostatic pathway [15]. Fibrinogen is converted into fibrin, a final product of hemostatic system, through the proteolytic effect of thrombin [15]. Fibrinogen plays important roles in blood coagulation, cell-cell adhesion and the inflammatory response [15]. Elevated fibrinogen is a well-known predictor of cardiovascular events and an independent predictor of mortality in patients with chronic kidney disease [16]. Additionally, recent studies have suggested that elevated fibrinogen promotes cancer cell growth, progression and metastasis [17–21]. Furthermore, plasma fibrinogen levels have been associated with tumor size, tumor invasion and lymph node metastasis in patients with various cancers [15]. In advanced GC, elevated fibrinogen levels have been associated with metastasis and tumor progression [15, 22]. Moreover, it has been reported that preoperative plasma fibrinogen levels are a useful predictor of lymphatic and hematogenous metastasis in GC [22, 23].

Hyperfibrinogenemia is increasingly being recognized as an important risk factor influencing cancer development and outcome. However, few reports have investigated the use of fibrinogen as a prognostic biomarker for GC patient survival. The primary aim of this study was to evaluate the usefulness of preoperative serum fibrinogen (FBG) as a biomarker for predicating tumor progression and survival of patients with GC. Additionally, we investigated the effects of multiple risk factors on the survival of GC patients.

## Methods

### Patients

This retrospective study was approved by the institutional review board of Harbin Medical University Cancer Hospital in China. The medical records of 1196 GC patients who were treated in the hospital between 2005 and 2007 were reviewed. The inclusion criteria for this study were: 1) age > 21 years; 2) histologically confirmed GC; and 3) GC treated via gastrectomy with D1, D2 or more extended lymph node dissection. Exclusion criteria included: 1) 3 months or less of follow-up data; 2) no preoperative plasma fibrinogen level data; 3) acute or chronic inflammatory diseases, coagulation disorders, chronic renal failure and acute or chronic liver disease; and 4) orally administered anticoagulation therapy.

For all patients enrolled in this study, we collected all data concerning patient demographics (age, sex and family history), clinicopathological characteristics, comorbidities, FBG levels, carcinoembryonic antigen (CEA) levels, Carbohydrate antigen 19-9 (CA199) levels, operative factors (type of gastrectomy, extent of lymph node dissection, operation time, intraoperative blood loss and transfusion requirements) and tumor characteristics (location, size, gross and pathological morphology, lymph node metastasis, distant metastasis, disease stage and median survival time, MST). Tumors were divided into two major categories based on histological characteristics: well-differentiated (papillary, well or moderately differentiated adenocarcinomas) and undifferentiated (poorly differentiated or undifferentiated adenocarcinomas, signet ring cell carcinomas and mucinous carcinomas) [11]. Surgical GC specimens were confirmed histologically. Gastrectomy and other operational procedures and reconstruction techniques were performed based on standardized methods that have been previously described [15]. Most of the radical lymphadenectomy means undergoing D2 lymph node dissection in our research.

### Fibrinogen, CEA and CA 19-9 measurements

Preoperative plasma fibrinogen, serum CEA and CA199 levels were examined in samples obtained from patients before breakfast within 7 days prior to surgery. Plasma fibrinogen levels were determined using the Clauss method and the Dimension Vista System (Siemens, Berlin, Germany) according to the manufacturer’s instructions. Normal preoperative plasma fibrinogen levels were defined as being between 2.0 and 4.0 g/L [24]. Serum fibrinogen concentrations between 1.5 and 4.0 g/L were considered normal, and concentrations of 4.0 g/L or above were considered hyperfibrinogenemic.

### Follow-up

Post-surgical outcomes for the entire cohort were followed for up to 5 years or until death. For the first 2 years after surgery, follow up examinations of all patients were performed once every 3 months. After 2 years, follow up examinations were performed every 6 months. The 6-month follow up examinations continued for up to 5 years. During the follow up examinations, physical examinations, laboratory tests, imaging and endoscopy were performed.

### Statistical analysis

Patient baseline characteristics were expressed as frequencies and percentages. The cutoff value of 4.0 g/L FBG was used to divide patients into low-and high-level FBG groups. Values are reported as means ± standard deviation (SD). Variables recorded for the patient groups were compared using the chi-squared test, Mann–Whitney U-test or Kruskal-Wallis test, as appropriate. Survival analysis (overall survival, OS) was performed using the Kaplan-Meier method, and comparisons between groups of interest were performed using the Log-rank test. The Cox regression model was used in a multivariate fashion to investigate the effects of selected confounding factors on the relationship between survival time and clinical characteristics. The results were presented in terms of the median survival time and hazard ratio (HR) with corresponding 95 % confidence intervals (CI). To determine the best cutoff point for patient survival prediction using FBG levels, receiver operating characteristic (ROC) curve analysis was performed for pre-treatment FBG levels, and an area under the curve (AUC) with a 95 % confidence interval (CI) was derived. A *p* value < 0.05 was considered statistically significant. All analyses were performed using Statistical Analysis System (SAS Institute, Cary, NC) software.

## Results

### Baseline patient characteristics

This study included 1196 GC patients, 885 (74.0 %) male and 311 (26.0 %) female. Patient demographics and clinicopathological characteristics are summarized in Table 1. Seventy-two patients (6.0 %) were younger than 40 years of age, 772 patients (64.6 %) were between 40 and 65 years of age and 352 patients (29.4 %) were older than 65 years of age. A majority of the patients (52.1 %) had BMIs less than 18.5 kg/m^2^, and 117 patients (9.8 %) had various comorbidities. Tumor sizes greater than 5 cm were present in 61.4 % of patients, and in 63.8 % of the patients, the tumors were located in the lower regions of the stomach. Tumor involvement of multiple organs was present in 4.6 % of patients.

**Table 1:** Baseline patient characteristics

Factor	Variable	No. of patients (%)
	Total number of patients	1196 (100.0)
Patient-related	Gender	
	Male	885 (74.0)
	Female	311 (26.0)
	Age (years)	
	<40	72 (6.0)
	40–65	772 (64.6)
	≥ 65	352 (29.4)
	Body mass index (kg/m^2^)	
	<18.5	623 (52.1)
	18.5–20	93 (7.8)
	20–24	284 (23.7)
	≥ 24	196 (16.4)
	Comorbidity	
	No	1079 (90.2)
	Yes	117 (9.8)
	Family history	
	No	960 (80.3)
	Yes	236 (19.7)
	Fibrinogen (g/L)	
	≤ 4	887 (78.3)
	>4	246 (21.7)
	Carcinoembryonic antigen	
	<5	348 (77.9)
	≥ 5	99 (22.1)
	Carbohydrate antigen 19-9	
	<37	1103 (92.2)
	≥ 37	93 (7.8)
Tumor-related	Tumor location	
	Lower	762 (63.8)
	Upper	184 (15.4)
	Medium	249 (20.8)
	Tumor size (cm)	
	<5	461 (38.6)
	≥ 5	735 (61.4)
	Multi-organ involvement	
	No	1141 (95.4)
	Yes	55 (4.6)
	Multifocal	
	No	1079 (97.6)
	Yes	27 (2.4)
	Gross morphology	
	Flat	142 (11.9)
	Uplift	137 (11.4)
	Ulcerative	146 (12.2)
	Infiltration ulcerative	702 (58.7)
	Diffuse infiltration	69 (5.8)
	T stage	
	1	195 (16.3)
	2	180 (15.1)
	3	395 (33.0)
	4	426 (35.6)
	N stage	
	N0	452 (37.8)
	N1	196 (16.4)
	N2	244 (20.4)
	N3	304 (25.4)
	Metastasis	
	No	1121 (93.7)
	Yes	75 (6.3)
	TNM	
	1	205 (17.1)
	2	318 (26.6)
	3	599 (50.1)
	4	74 (6.2)
	Differentiation	
	Poor	513 (42.9)
	Medium	246 (20.6)
	Signet ring	34 (2.8)
	Mucus	47 (3.9)
	Papillary	8 (0.7)
	Mixed or other	348 (29.1)
Operation-related	Operation time (in min.)	
	≤ 150	616 (51.5)
	>150	580 (48.5)
	Mode of lymph node dissection	
	Radical	856 (71.6)
	Non-radical	250 (20.9)
	Palliative	90 (7.5)
	Cleared lymph node number	
	<15	667 (55.8)
	≥ 15	529 (44.2)
	Intraoperative blood loss (ml)	
	≤ 200	977 (81.7)
	>200	219 (18.3)
	Blood transfusion	
	No	901 (75.3)
	Yes	295 (24.7)

More than half of the patients enrolled in this study had advanced GCs. Seven-hundred and two patients (58.7 %) had tumors that exhibited ulcerative infiltration, and 42.9 % of patients had poor tumor differentiation. The disease stage at the time of the GC diagnosis was Stage 1 in 195 (16.3 %) patients, Stage 2 in 180 (15.1 %) patients, Stage 3 in 395 (33.0 %) patients and Stage 4 in 426 (35.6 %) patients. Lymph node metastasis was N0 in 452 (37.8 %) patients, N1 in 196 (16.4 %) patients, N2 in 244 (20.4 %) patients and N3 in 304 (25.4 %) patients. Metastasis was present in 75 (6.3 %) patients, and, correspondingly, no metastasis was detected in 1121 (93.7 %) of the patients.

Most patients (71.6 %) underwent radical lymph node dissection, and only a small proportion of the patients underwent non-radical (20.9 %) or palliative (7.5 %) lymph node dissection. Most patients experienced intraoperative blood loss of less than 200 ml (81.7 %), and only 24.7 % of the patients received postoperative blood transfusions. The median survival duration was 55.62 months. Blood biomarker detection indicated that most patients had normal levels of serum FBG (78.3 %), CEA (77.9 %) and CA199 (92.2 %; Table 1).

### Univariate analysis of prognostic factors

In this study, the MST of GC patients after surgery was 41.9 ± 32.4 months. To assess the prognostic factors affecting patient survival, we conducted univariate analysis of the MST in relation to the various demographic and clinicopathological factors of the enrolled patients. The univariate analysis indicated that gender, age, BMI and the presence of comorbidities were not risk factors for survival (all *p* > 0.05). However, other demographic and clinicopathological factors were significantly associated with patient survival. These factors included a family history of GC (HR 0.8; *p* <0.05), the mode of lymph node dissection (Non-radical, HR 4.74, *p* < 0.001; Palliative, HR 12.21, *p* < 0.001), tumor size (≥ 5 cm, HR 3.29, *p* < 0.001), tumor location (Upper, HR 1.83, *p* < 0.001; Medium, HR 2.03, *p* < 0.001), poor tumor differentiation (HR 1; *p* < 0.001), histological classification of the tumor (*p* < 0.001), extent of tumor invasion (*p* < 0.001), number of metastatic lymph nodes (*p* < 0.001), advanced disease stages (HR 50.32, *p* < 0.001), extended duration of the operation (> 150 min, HR 1.17, *p* < 0.001), higher operative bleeding volume (> 200 ml, HR 1.61, *p* < 0.001), postoperative transfusion (HR 1.64, *p* < 0.001), FBG levels (> 4.0 g/L, HR 1.78, *p* < 0.001), CEA levels (≥ 5.0 g/L, HR 1.82, *p* < 0.001) and CA 19-9 levels (≥ 37.0 g/L, HR 1.84, *p* < 0.001). The results of the univariate analysis are shown in Table 2.

**Table 2:** Univariate analysis of risk factors affecting patient survival

Factor	Variable	Survival months (means ± SE)	HR (95 % CI)	*p* value
	Total	41.9 ± 32.4		
Patient-related	Gender			
	Male	41.0 ± 32.1	1	
	Female	44.4 ± 33.0	0. 87 (0.73–1.03)	0.1074
	Age (years)			
	<40	47.1 ± 32.4	1	
	40–65	42.4 ± 32.7	1.15 (0.83–1.60)	0.4107
	≥ 65	39.6 ± 31.6	1.32 (0.94–1.87)	0.1123
	Body mass index (kg/m^2^)			
	<18.5	42.1 ± 33.5	1	
	18.5–20	36.5 ± 29.2	1.13 (0.86–1.49)	0.3774
	20–24	42.5 ± 30.9	0.91 (0.75–1.10)	0.3137
	≥ 24	42.8 ± 32.1	0.95 (0.77–1.17)	0.6183
	Comorbidity			
	No	41.6 ± 32.1	1	
	Yes	43.9 ± 34.6	0.93 (0.72–1.21)	0.5909
	Family history			
	No	41.3 ± 32.5	1	
	Yes	44.4 ± 32.0	0.80 (0.66–0.98)	0.0286
	Fibrinogen (g/L)			
	≤ 4	45.7 ± 32.6	1	
	>4	30.8 ± 28.9	1.78 (1.49–2.11)	< 0.0001
	Carcinoembryonic antigen			
	<5	43.4 ± 29.3	1	
	≥ 5	31.7 ± 29.1	1.82 (1.38–2.38)	< 0.0001
	Carbohydrate antigen 19-9			
	<37	43.2 ± 32.6	1	
	≥ 37	26.4 ± 24.6	1.84 (1.44–2.35)	< 0.0001
Tumor-related	Tumor location			
	Lower	47.4 ± 32.7	1	
	Upper	34.2 ± 30.3	1.83 (1.50–2.23)	< 0.0001
	Medium	30.7 ± 28.7	2.03 (1.70–2.42)	< 0.0001
	Tumor size (cm)			
	<5	58.5 ± 30.0	1	
	≥ 5	31.5 ± 29.4	3.29 (2.75–3.94)	< 0.0001
	Multi-organ involvement			
	No	42.7 ± 32.3	1	
	Yes	24.3 ± 28.5	2.27 (1.68–3.07)	< 0.0001
	Multifocal			
	No	44.4 ± 32.0	1	
	Yes	55.8 ± 28.5	0.67 (0.38–1.19)	0.1721
	Gross morphology			
	Flat	34.0 ± 36.0	1	
	Uplift	47.4 ± 33.1	0.56 (0.41–0.76)	0.0003
	Ulcerative	55.0 ± 32.3	0.42 (0.31–0.59)	< 0.0001
	Infiltration ulcerative	40.8 ± 30.8	0.71 (0.57–0.90)	0.0037
	Diffuse infiltration	30.4 ± 29.1	1.09 (0.77–1.55)	0.6112
	T stage			
	1	44.8 ± 37.0	1	
	2	64.1 ± 27.4	0.35 (0.25–0.51)	< 0.0001
	3	41.2 ± 30.4	1.20 (0.94–1.52)	0.1397
	4	31.7 ± 28.8	1.80 (1.42–2.27)	< 0.0001
	N stage			
	N0	52.6 ± 33.4	1	
	N1	53.0 ± 31.2	1.12 (0.87–1.45)	0.3837
	N2	35.6 ± 29.7	2.36 (1.91–2.92)	< 0.0001
	N3	23.8 ± 23.0	3.72 (3.06–4.53)	< 0.0001
	Metastasis			
	No	44.1 ± 32.2	1	
	Yes	8.7 ± 7.7	5.75 (4.48–7.38)	< 0.0001
	TNM			
	1	70.2 ± 25.2	1	
	2	56.4 ± 29.6	4.45 (2.80–7.07)	< 0.0001
	3	28.6 ± 26.5	15.14 (9.76–23.49)	< 0.0001
	4	8.8 ± 7.7	50.32 (30.71–82.46)	< 0.0001
	Differentiation			
	Poor	33.0 ± 31.1	1	
	Medium	55.0 ± 30.3	0.38 (0.30–0.48)	< 0.0001
	Signet ring	47.5 ± 30.5	0.49 (0.30–0.82)	0.0058
	Mucus	49.8 ± 31.5	0.58 (0.39–0.86)	0.0064
	Papillary	45.1 ± 32.6	0.54 (0.20–1.44)	0.2174
	Mixed or other	44.0 ± 32.2	0.64 (0.53–0.76)	< 0.0001
Operation-related	Operation time (min.)			
	≤ 150	43.3 ± 32.9	1	
	>150	40.3 ± 31.8	1.17 (1.01–1.36)	0.0361
	Mode of lymph node dissection			
	Radical	52.2 ± 30.8	1	
	Non-radical	18.8 ± 20.4	4.74 (4.00–5.62)	< 0.0001
	Palliative	7.2 ± 6.8	12.21 (9.52–15.67)	< 0.0001
	Cleared lymph node number			
	<15	40.5 ± 33.2	1	
	≥ 15	43.6 ± 31.3	0.81 (0.70–0.95)	0.0072
	Intraoperative blood loss (ml)			
	≤ 200	44.1 ± 32.6	1	
	>200	31.7 ± 29.4	1.61 (1.35–1.93)	< 0.0001
	Blood transfusion			
	No	44.6 ± 32.3	1	
	Yes	33.4 ± 31.0	1.64 (1.39–1.93)	< 0.0001

### Multivariate analysis of prognostic factors

To identify the independent risk factors that could be used to predict MST, we performed multivariate analysis of prognostic factors and MST using the Cox proportional hazards model. Factors included in the multivariate analysis included the mode of lymph node dissection, the presence of multi-organ involvement, the stage of the disease, FBG levels, CA199 levels and tumor differentiation. The analysis indicated that several of these independent prognostic factors were significantly associated with poor GC patient survival. The significantly associated factors included non-radical lymph node dissection (HR 2.66; *p* < 0.0001; 95 % CI 2.20–3.22), palliative lymph node dissection (HR 16.97; *p* < 0.0001; CI 9.07–31.72), multi-organ involvement (HR 2.06; *p* < 0.0001; CI 1.50–2.84), advanced TNM stages, poor tumor differentiation, higher FBG levels (HR 1.36; *p* = 0.0008; CI 1.14–1.62) and higher CA199 levels (HR 1.39; *p* = 0.0115; CI 1.08–1.79; Table 3). Notably, the T stage was not significantly associated with poor prognosis.

**Table 3:** Multivariate analysis of risk factors affecting patient survival

Variable	HR (95 % CI)	*p*
Lymph node dissection (radical vs. non-radical)	2.66 (2.20–3.22)	< 0.0001
Lymph node dissection (radical vs. palliative)	16.97 (9.07–31.72)	< 0.0001
Multi-organ involvement (yes vs. no)	2.06 (1.50–2.84)	< 0.0001
T stage (2 vs. 1)	0.72 (0.44–1.16)	0.1744
T stage (3 vs. 1)	1.14 (0.74–1.78)	0.5501
T stage (4 vs. 1)	1.33 (0.86–2.04)	0.1965
N stage (1 vs. 0)	1.37 (0.96–1.97)	0.0851
N stage (2 vs. 0)	2.44 (1.61–3.70)	< 0.0001
N stage (3 vs. 0)	3.22 (2.09–4.94)	< 0.0001
Metastasis (yes vs. no)	81.97 (10.49–640.70)	< 0.0001
TNM stage (2 vs. 1)	3.00 (1.71–5.26)	0.0001
TNM stage (3 vs. 1)	3.37 (1.68–6.76)	0.0006
TNM stage (4 vs. 1)	0.08 (0.01–0.60)	0.0148
Differentiation (medium and good vs. poor)	0.65 (0.51–0.83)	0.0006
Differentiation (mucous vs. poor)	0.53 (0.34–0.83)	0.0054
Differentiation (papillary vs. poor)	0.94 (0.35–2.55)	0.9075
Differentiation (mixed and others vs. poor)	0.90 (0.75–1.09)	0.2895
Differentiation (Signet Ring vs. poor)	0.90 (0.54–1.49)	0.6717
Fibrinogen (g/L) (>4 vs. ≤4)	1.36 (1.14–1.62)	0.0008
Carbohydrate antigen 19-9 (≥37 vs. < 37)	1.39 (1.08–1.79)	0.0115

### The diagnostic value of serum FBG levels

**Fig. 1:**
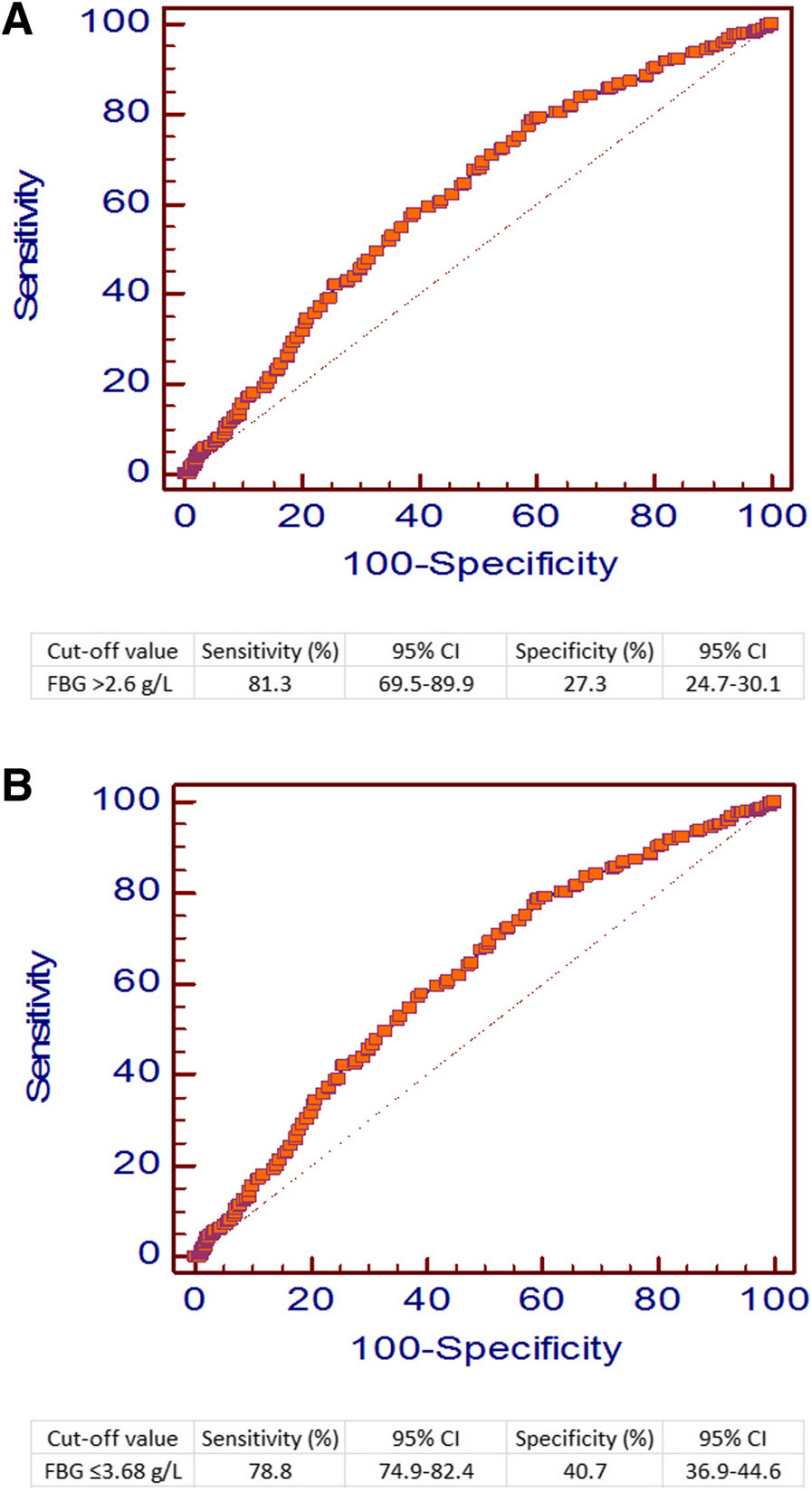
*Receiver operating characteristic (ROC) curve analysis performed using pre-operational fibrinogen (FBG) levels to determine the best cutoff point for predicting the survival of gastric cancer patients.* **a** When FBG cutoff value was set > 2.6 g/L, the sensitivity was 81.3 %, the 95 % confidence interval (CI) was 69.5–89.9; the specificity was 27.3 % and the 95 % CI was 24.7–30.1; **b** When the FBG cutoff value was set ≤ 3.68 g/L, the sensitivity was 78.8 %, the 95 % CI was 74.9–82.4; the specificity was 40.7 % and the 95 % CI was 36.9–44.6

To determine the diagnostic value of serum FBG levels, ROC curve analysis was performed and an area under the curve (AUC) with a 95 % confidence interval (CI) was derived. When the FBG cut-off value was > 2.6 g/L, the sensitivity was 81.3 % (95 % CI: 69.5–89.9) and the specificity was 27.3 % (95 % CI: 24.7–30.1). When the FBG cut-off value was ≤ 3.68 g/L, the sensitivity was 78.8 % (95 % CI: 74.9–82.4) and the specificity was 40.7 % (95 % CI: 36.9–44.6). Therefore, we set an FBG cut off value of 4.0 g/L in this study (Fig. 1).

### Serum FBG levels are positively correlated with tumor progression and metastasis

The multivariate analysis described above indicated that elevated serum FBG levels were an independent prognostic factor of poor patient survival. To investigate the correlations between serum FBG and the disease stage of the tumor, we performed Chi-square analysis of FBG levels in patients with different T, N and pathological stages. We observed significant differences in the serum FBG levels of patients with different T stages (*F* = 11.94, *p* < 0.0001), N stages (*F* = 4.93, *p* = 0.0021) and pathological stages (*F* = 16.13, *p* < 0.0001). Additionally, correlation analysis indicated that serum FBG levels were positively correlated with patient T stages (t = 4.63, *p* < 0.0001), N stages (t = 3.83, *p* = 0.0001) and pathological stages (t = 6.50, *p* < 0.0001; Table 4).

**Table 4:** Correlations between serum FBG and tumor TNM stage

	No. of patients (%)	Chi-square test	Fibrinogen (g/L) (means ± SE)	Regression analysis
T stage		*F* = 11.94*, p < 0.*0001		t = 4.63, *p* < 0.0001
1	195 (16.3)		3.19 ± 0.97	
2	180 (15.1)		2.94 ± 0.85	
3	395 (33.0)		3.33 ± 1.01	
4	426 (35.6)		3.45 ± 0.99	
N stage		*F* = 4.93*, p =* 0.0021		t = 3.83, *p* = 0.0001
N0	452 (37.8)		3.17 ± 0.94	
N1	196 (16.4)		3.24 ± 1.02	
N2	244 (20.4)		3.37 ± 1.00	
N3	304 (25.4)		3.44 ± 1.00	
Pathological stage		*F* = 16.13*, p < 0.*0001		t = 6.50, *p* < 0.0001
1	205 (17.1)		2.94 ± 0.89	
2	318 (26.6)		3.18 ± 0.95	
3	599 (50.1)		3.46 ± 1.00	
4	74 (6.2)		3.38 ± 1.02	

### Serum FBG levels are positively correlated with survival of patients

**Fig. 2:**
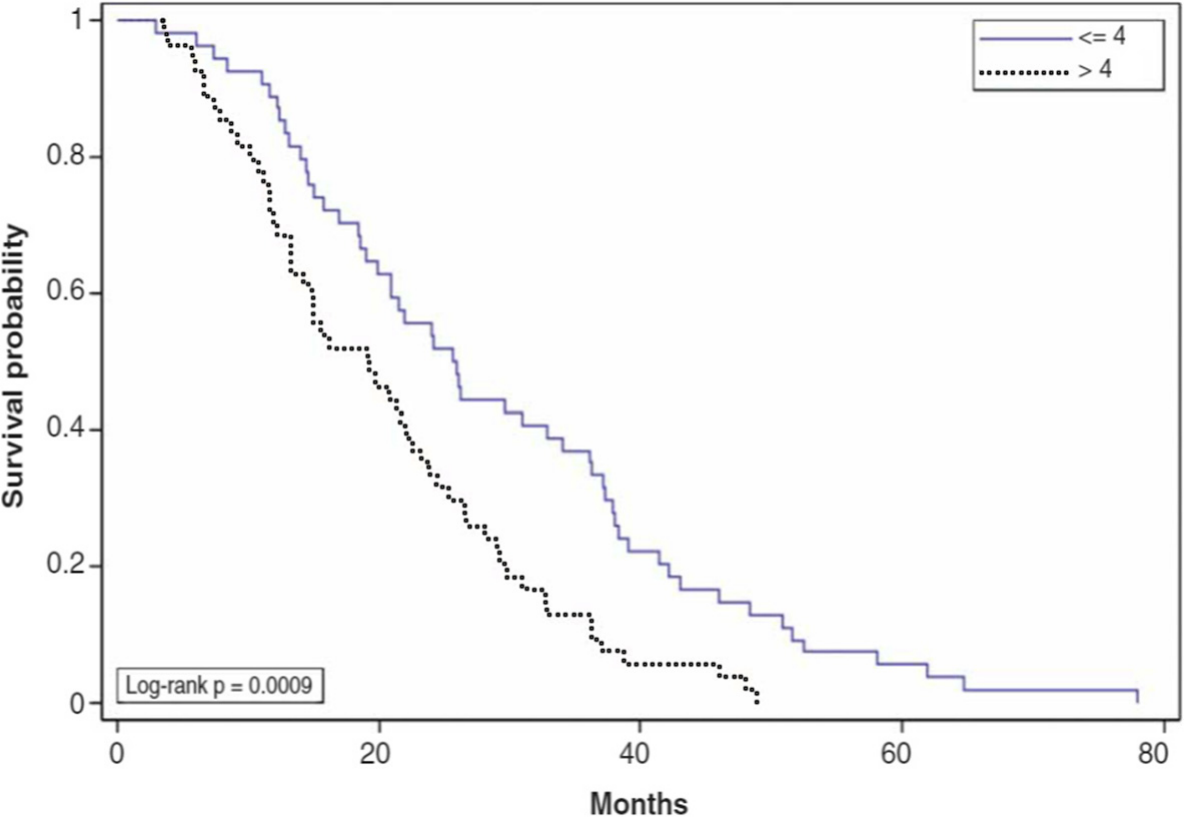
*Overall survival according to pre-operational serum fibrinogen (FBG) levels.* Gastric cancer patients with FGB > 4.0 g/L have a lower overall survival rate when compared with patients with FGB < 4.0 g/L *(p =* 0.0009)

To study the correlations between serum FBG levels and patient survival, we compared the overall survival rates of patients with normal serum FBG levels with the overall survival rates of patients with high serum FBG levels. We observed that, after surgery, GC patients in the high FBG level group (> 4.0 g/L) had a significantly lower survival rate when compared with the normal FBG level group (≤ 4.0 g/L; *p* = 0.0009; log-rank test; Fig. 2).

## Discussion

In this comprehensive retrospective study of the risk factors influencing GC patient survival after gastrectomy, we analyzed patient-related, tumor-related and operation-related demographic and clinicopathological data from 1196 patients who were treated for operable GC in Harbin Medical University Cancer Hospital between 2005 and 2007. The results of our study indicate that many factors influence the survival rates and survival time of post-surgical GC patients. However, the most important finding reported by this study is that serum FBG levels are positively correlated with the progression and metastasis of GC. Thus, our results indicate that FBG is an independent risk factor that can be used to predict GC patient survival. Additionally, our results confirm the importance of other well-known tumor-related and operation-related factors.

FBG is an acute-phase protein that regulates clotting and fibrinolysis, and hyperfibrinogenemia has frequently been linked with a number of malignancies [20, 21, 25]. It is thought that the link between hyperfibrinogenemia and cancer may be related to the systemic activation of the clotting system observed in many cancer patients [26]. Two possible mechanisms underlying this relationship include cancer cell driven stimulation of FBG levels by direct activation of the clotting response to produce procoagulant factors (including FBG), and indirect stimulation of mononuclear cells to secrete these factors [27]. A strong correlation between FBG and enlarged tumor size, increased tumor growth, increased metastatic potential and poor prognosis in various cancers is increasing being recognized [17–21, 25]. Preston et al. [28] reported that fibrinogen production was elevated in pancreatic adenocarcinoma patients. Shen et al. [20] examined FBG levels in 567 patients with operable non-small cell lung cancer and reported that serum fibrinogen was an independent prognostic factor. Patients with hyperfibrinogenemia in the Shen et al. study had a higher risk of disease progression and mortality when compared with patients that had normal fibrinogen levels [20]. Tanaka et al. [25] reported that preoperative plasma fibrinogen levels higher than 450 mg/dL were an independent risk factor of subsequent tumor recurrence and cancer-specific survival in patients with localized upper tract urothelial carcinomas. Additionally, Tanaka et al. demonstrated that high plasma fibrinogen levels predicted worse pathological features and positive lymphovascular invasion [20]. Lee et al. [15, 29] reported that tumor size, tumor depth, tumor extent, lymph node metastasis and poor patient survival were positively correlated with preoperative plasma fibrinogen levels in advanced GC.

To determine the diagnostic value of serum FBG levels, ROC curve analysis was performed and an area under the curve (AUC) with a 95 % confidence interval (CI) was derived. We set an FBG cut off value of 4.0 g/L. When the FBG cut-off value was ≤ 3.68 g/L, the sensitivity was 78.8 % and the specificity was 40.7 %. A previous study reported that when plasma fibrinogen levels > 402 mg/dL were defined as hyperfibrinogenemia based on ROC curve analysis, fibrinogen concentration had a PPV of 92.73 % [30].

Our current study supports the hypothesis that plasma FBG levels are positively correlated with the advanced T stages, N stages and pathological stages of GC. Furthermore, our results indicate that FBG is an important independent factor that influences the survival of GC patients. These findings are consistent with the findings of previous studies [15, 29] and indicate that elevated fibrinogen levels are associated with advanced GC metastasis and tumor progression [15, 22]. Therefore, serum fibrinogen may be a useful biomarker for the identification of patients with clinically advanced GC. Additionally, preoperative plasma fibrinogen level is a useful predictor of adjacent organ involvement in advanced GC patients [15]. It may also be useful to monitor serum FBG levels during ongoing patient management. This report does not discuss patient management, and a future manuscript will discuss the effects of various therapies on fibrinogen levels in patients with gastric cancer. Although serum FBG is a valuable marker, levels could be affected by underlying disease, such as, for example, in cardiac infarction.

Hyperfibrinogenemia may help provide favorable conditions for cancer cell metastasis via the lymphatic system [22], and preoperative plasma fibrinogen levels are a useful predictor of lymphatic as well as hematogenous metastasis in GC [22, 23]. However, the molecular mechanisms through which fibrinogen promotes tumor metastasis remain unclear. Fibrinogen is a dimeric molecule and has various integrin and non-integrin binding motifs. Because cancer cells usually produce an elevated number of fibrinogen receptors (eg α5β1, αvβ3 integrins and the ICAM-1 molecule), some scholars have proposed that fibrinogen may facilitate tumor and host cell interactions, thus facilitating tumor cell metastasis. Another possible metastasis promoting mechanism is the fibrinogen-facilitated formation of large tumor cell aggregates with platelet αIIbβ3 integrin receptors. By forming these large aggregates, cancer cells are able to avoid detection by the innate immune system and, thus, the metastatic potential of the aggregated cells is increased [23, 31, 32]. A third possible metastasis promoting mechanism is caused by a positive feedback loop between cancer induced inflammation and fibrinogen levels. The increased systemic inflammatory response caused by cancer progression greatly enhances the levels of fibrinogen, and the elevated fibrinogen, in turn, promotes cancer cell metastasis [22].

In addition to FBG levels, we also analyzed the levels of CEA and CA19-9 in GC patients. Both CEA and CA19-9 are well known GC biomarkers [3, 33]. In the present study, we confirmed that patients with higher CEA and CA19-9 levels had a greatly reduced survival time. According to the multivariate analysis conducted in our study, elevated serum CA19-9 levels were an independent predictor of poor survival in GC patients, but CEA levels were not. However, our results indicate that FBG levels may be a better GC biomarker when compared with CEA and CA19-9 levels.

Interestingly, univariate analysis indicated that patient related factors, including age, sex, BMI and comorbidity, had no effect on patient survival. The only exception was a family history of GC. Regarding patient age, the results of our analysis differ from a previous report by Liang et al. [34]. In the Liang et al. study, the authors claimed that patient age greater than 70 years was an independent prognostic factor for GC after gastrectomy, and elderly patients (OS: 22.0 %) had a significantly lower 5-year OS rate when compared with younger (OS: 36.6 %) and middle-aged patients (OS: 38.0 %). Inokuchi et al. [35] reported that comorbidity predicted post-gastrectomy complications in patients with large GC tumors. However, our results indicate that patients both with and without comorbidities have similar median survival times. The differences between the results of our current study and the Inokuchi et al. report is likely due to differences in the severity of comorbidities in each patient population.

We found that patients with a family history of GC have shorter survival times when compared with patients without any family history of GC (41.3 months vs. 44.4 months). This finding is in accordance with a report from Liang et al. [36]. In the Liang et al. report, they compared the clinicopathological characteristics of 91 patients with familial GC (FGC) and 293 patients with sporadic GC (SGC). They reported that the 5-year overall survival rate in the FGC patients was significantly lower than that in the SCG patients (25.6 % vs. 38.9 %, *p* = 0.001). FGC was correlated with poor GC differentiation and prognosis.

Various studies have suggested that tumor size not only determines the extent of disease, tumor metastasis and invasion, but that it also predicts patient survival [13–15]. Tumor size has been reported to be an independent prognostic factor, and a modified TNM system based on tumor size accurately predicts patient survival [11]. In the present study, univariate analysis suggested that patients with tumors larger than 5 cm had a shorter survival time when compared with patients that had tumors smaller than 5 cm. Although there were significant differences, tumor size was not found to be an independent predictor of patient survival by our multivariate analysis. Our study agrees with the report from Lu et al. [10]. However, other reports have suggested that tumor size is an independent predictor of patient survival [37]. In addition to tumor size, tumor location is another factor that may influence patient survival. We found that the tumors located in the upper and medium gastric areas were associated with a poor patient prognosis. However, multivariate analysis did not find that tumor location was an independent factor. In contrast with tumor size and location, multivariate analysis indicated that poor differentiation, deep invasion and lymph node metastasis were independent factors.

Lymph node metastasis and advanced TNM stage are considered the most important factors for the prediction of recurrence and survival in GC patients [4–9]. Lymph node metastases are observed in more than 50 % of GC patients at the time of diagnosis. The 5-year survival rate of patients with lymph node metastasis is reported to be approximately 30 % [9]. The analysis reported here confirms that lymph node metastasis is a critical prognostic factor in GC patients, and that lymph node metastasis correlates strongly with shorter survival time and poor patient prognosis. In addition to lymph node metastasis, we also found that GC invasion of other organs was an independent risk factor for survival.

Surgery is an important factor in the treatment of GC patients, and operation related factors are another type of important risk factor associated with patient mortality and survival. Operational factors that we examined in the present study include the mode of lymph node dissection, operational time, intraoperative blood loss and transfusion treatment. Our analysis indicated that radical lymph node dissection is an independent favorable factor for GC patient survival. Patients that received radical lymph node dissection survived as long as 52 months, while those who did not receive the therapy survived less than one and half a years. A previous report suggested that intraoperative blood loss was an independent prognostic factor for GC patients who had undergone curative resection [12]. Reducing intraoperative blood loss improved the long-term outcome of GC patients treated with curative gastrectomy [12]. In the present study, we did not conduct multivariate analysis on intraoperative blood loss; therefore, we are unable to conclude that intraoperative blood loss is an independent risk factor for survival. However, we do confirm that certain operational factors (operational time more than 150 min, blood loss more than 200 ml and insufficient or delayed blood transfusion) are associated with GC patient survival. Notably, the receipt of a blood transfusion can significantly shorten survival time.

Although this is a comprehensive study related to FBG and other prognostic factors in GC patients, we did not conduct further analysis of risk factors that could affect serum FBG levels.

## Conclusions

Preoperative hyperfibrinogenemia is correlated with GC progression, and FBG levels are an independent risk factor of GC patient survival in patients with operable GC. Additionally, serum fibrinogen is a useful biomarker for patients with clinically advanced GC. Furthermore, tumor and operative factors (non-radical lymph node dissection, multi-organ involvement, advanced TNM stages and poor tumor differentiation) and CA199 levels are independent prognostic factors that are also associated with poor GC patient survival after gastrectomy.

## Abbreviations

ASR, age standardized rates; AUC, area under the curve; CA199, carbohydrate antigen 19-9; CEA, carcinoembryonic antigen; CI, confidence interval; CI, confidence intervals; FBG, fibrinogen; FGC, familial GC; GC, gastric cancer; HR, hazard ratio; MST, median survival time; OS, overall survival; ROC, receiver operating characteristic; SD, standard deviation; SGC, sporadic GC
